# 

**Published:** 1861-02

**Authors:** 


### CONTENTS FOR FEBRUARY, 1861.

### ORIGINAL COMMUNICATIONS.

Abstract of a Paper on the Influence of Alcoholic Drinks, on the Development and Progress of Tuberculosis. By N. S. DAVIS, M. D. .......... 65
Mercy Hospital. Service of E. ANDREWS, M. D., A Clinical Lecture upon Facial Neuralgia .......... 71
Case of Prolapse of Funis and Craniotomy. By F. N. SMITH, M. D. .......... 76
An Essay on Diphtheria. By HENRY W. DAVIS, M. D. .......... 79

### SELECTIONS.

Ergot; its Natural History and Uses as a Therapeutic Agent .......... 93
On Pelvic Measurements. By W. H. BYFORD, M. D .......... 112
Influence of Tobacco Smoking on Public Health .......... 114
Treatment of Diphtheria, Inflammatory Angina and Croup, by the Perchloride of Iron. 116
The Smithsonian Institution .......... 117
Position a remedy for Stertor .......... 117
Iodide of Amonium .......... 117

### EDITORIAL.

Report of the Committee on Medical Education, presented to the Academy of Medical Sciences, Jan. 4th, 1861 .......... 118
Anesthetics .......... 122
Arseniate of Gold in the Treatment of Cancer .......... 126
Aromatic Sulphuric Acid in Treatment of Tapeworm .......... 126
Chloroform in Congestive Chills .......... 127
Digitalis in Delirium Tremens .......... 127
American Medical Association .......... 127
Summer Course of Medical Instruction .......... 127

